# Psychological Distress and Health-Related Quality of Life in Ménière’s Disease: A Comparative Study in the Portuguese Population

**DOI:** 10.3390/jcm15103919

**Published:** 2026-05-19

**Authors:** Diogo Ribeiro, Tânia Martins, André Oliveira, Sara Simões Dias, Cristina Caroça

**Affiliations:** 1 CHRC, Comprehensive Health Research Centre—Advanced Human Genetics, NOVA Medical School, Faculdade de Ciências Médicas, Universidade NOVA de Lisboa, 1169-056 Lisbon, Portugal; cristinacaroca@icloud.com; 2 Cintramédica—Clínica de Imagem Médica, 2710-437 Sintra, Portugal; aloliveira11.porto@gmail.com; 3 Hospital CUF Tejo, 1350-352 Lisbon, Portugal; taniadfmartins87@gmail.com; 4 Center for Innovative Care and Health Technology (ciTechCare), Escola Superior de Saúde, Politécnico de Leiria, 2411-901 Leiria, Portugal; sara.dias@ipleiria.pt

**Keywords:** Ménière’s disease, tinnitus, dizziness, hearing loss, psychological distress, health-related quality of life, SF-36, EQ-5D, anxiety, depression

## Abstract

**Background/Objectives**: Ménière’s disease (MD) is a chronic inner-ear disorder characterized by vertigo, tinnitus, and fluctuating sensorineural hearing loss (SNHL) that impairs health-related quality of life (HRQoL) and is frequently accompanied by psychological distress. This study aims to quantify psychological distress and generic HRQoL in patients with definite MD compared with age- and sex-matched general-population controls and to examine the associations between hearing loss (HL) severity, symptom-related handicap, and these outcomes within the MD cohort. **Methods**: In this observational cross-sectional study, 45 adults with definite MD were recruited from an otolaryngology clinic in Portugal and individually matched (1:3) by sex and 10-year age strata to 135 participants from the EpiDoC population cohort. HRQoL was assessed using the SF-36v2 and EQ-5D-3L, psychological distress using the HADS, and tinnitus- and dizziness-related handicap using the THI and DHI. HL severity was staged using the AAO-HNS (1995) criteria. **Results**: Patients with MD had higher anxiety and depression scores and nearly twice the prevalence of abnormal anxiety compared with controls. SF-36v2 scores were significantly worse in several role and psychosocial domains, with less bodily pain but similar physical functioning and general health. EQ-5D-3L revealed a lower utility index and higher pain/discomfort and anxiety/depression. Within the MD cohort, HL stage was only weakly related to distress and HRQoL. In contrast, higher THI and DHI scores were consistently associated with more severe anxiety and depression and lower SF-36v2 scores. In multivariable analysis, abnormal anxiety was strongly associated with moderate-to-severe tinnitus handicap (OR = 17.5), whereas higher depressive symptoms independently predicted moderate-to-severe dizziness handicap, with abnormal anxiety showing a strong but borderline association with the latter. **Conclusions**: MD confers clinically meaningful HRQoL decrements and a higher psychological distress burden than in the general population. Tinnitus- and dizziness-related handicap and psychological distress, particularly anxiety, rather than HL severity, are key multidisciplinary management targets. Systematic screening for anxiety and depression, regardless of audiometric stage, should be integrated into MD care pathways.

## 1. Introduction

Ménière’s Disease (MD) is a chronic inner-ear disorder characterized by the classic triad of episodic vertigo, tinnitus, and recurrent, fluctuating low- to medium-frequency sensorineural hearing loss (SNHL), often accompanied by aural fullness [1]. Despite recent advances in pathophysiology, MD remains challenging to manage due to uncertainties regarding its epidemiology, etiology, pathogenesis, and treatment [2,3].

The etiology of MD has traditionally been linked to histological findings of endolymphatic hydrops, a pathological hallmark characterized by excessive accumulation of endolymph, dilation of the cochlear duct, and increased pressure within the inner ear [4]. However, the cellular and molecular mechanisms underlying this phenomenon remain largely unknown [2,4,5]. Proposed theories include immune-mediated inflammation, genetic predisposition, anatomical variations, viral infections, vascular irregularities, and metabolic and endocrine imbalances [2,6,7,8,9].

Several professional societies have proposed diagnostic criteria for MD [1,10]. However, implementation differences have led to wide variations in reported frequencies across studies [1,10,11,12,13,14]. As a chronic, non-fatal condition, MD becomes more common with age and typically manifests in midlife, whereas pediatric cases are rare [3,15,16,17,18,19]. The disease appears to be slightly more prevalent in women and Caucasian populations, and bilateral involvement occurs in only a minority of patients. However, estimates vary depending on diagnostic stringency [17,20,21,22,23,24].

Building on this heterogeneous clinical profile, the course of MD is unpredictable and often accompanied by severe hearing loss (HL), aural fullness, tinnitus, vertigo, dizziness, vestibular drop attacks, and balance and gait impairments [3]. Consequently, patients frequently face substantial functional limitations due to psychological, physical, and cognitive impairments, markedly reducing health-related quality of life (HRQoL) [25,26,27]. However, the fluctuating and overlapping nature of HL, tinnitus, and vertigo often makes it difficult to determine which specific symptom contributes the most to the patient’s overall impairment and perceived quality of life (QoL) [26,28,29]. Despite this burden, relatively few studies have jointly quantified psychological distress and generic HRQoL in MD using validated instruments such as the Hospital Anxiety and Depression Scale (HADS), Short Form 36-Item Health Survey, version 2 (SF-36v2), and EuroQol 5-Dimension 3-Level questionnaire (EQ-5D-3L), particularly in comparison with population-based controls.

Psychological impairments can result from psychological distress, such as anxiety and depression, which, in turn, contribute to a decline in HRQoL [30,31,32]. According to the World Health Organization (WHO), anxiety disorders affect approximately 359 million people worldwide, and about 5–6% of adults live with depression, underscoring their substantial global burden [33,34,35]. Both conditions are more prevalent in women than in men and frequently co-occur with chronic somatic illnesses, resulting in reduced HRQoL and work capacity and increased healthcare use [33,34,35,36].

Weidt et al. showed that higher emotional distress and dizziness handicap are closely associated with poorer HRQoL on generic instruments, such as the SF-36 and EQ-5D, in patients with dizziness, highlighting the tight bidirectional link between vestibular symptoms and mental health [30]. In MD, several studies have reported that psychological distress is common and clinically relevant, with elevated anxiety, depression, and stress compared with healthy controls or non-vestibular patient groups [31,32,37]. Orji further highlighted that personality traits, life stress, and coping styles can modulate the intensity and intrusiveness of vertigo and tinnitus, thereby amplifying their effects on patients’ daily functioning and psychosocial well-being [31]. Consistently, EQ-5D health utility values are substantially lower than in the general population, and much of this reduction can be explained through vestibular and audiological symptoms, self-reported disabilities, and disease-specific handicap scores [38]. However, because EQ-5D mainly captures the overall burden rather than symptom-specific differences, current guidelines recommend combining generic HRQoL measures with disease-specific instruments such as the Tinnitus Handicap Inventory (THI) and Dizziness Handicap Inventory (DHI) [27].

In addition, HRQoL in MD is significantly affected by the classic symptomatic triad of physical impairments [27,28,38]. SNHL, which predominantly affects the low- to medium-frequency range, is typically recurrent and fluctuating, and impairs sound perception in one or both ears, contributing to communication difficulties and reduced HRQoL. [1,10,28,29,39]. Tinnitus, another key symptom of this triad, is the perception of sound in the ears or head without an external acoustic source, often fluctuating, and associated with cochlear dysfunction [1,40,41]. Vertigo, a hallmark episodic symptom of MD, is a specific form of dizziness characterized by a false sensation of rotational movement (spinning), typically lasting from 20 min to several hours, resulting from vestibular dysfunction [1].

Söderman et al. showed that HL and tinnitus have a greater impact on the psychosocial aspects of HRQoL, whereas vertigo mainly affects physical functioning [28]. Recent data further suggest that conventional pure-tone thresholds do not always parallel functional communication difficulties in MD, with some patients showing a marked mismatch between audiogram-based severity and speech recognition or perceived handicap, a phenomenon termed “functional audiometric dissociation” [5]. Similarly, studies in tinnitus cohorts have reported only weak correlations between audiometric loss and tinnitus distress, indicating that psychological and perceptual factors can drive substantial handicaps, even in the presence of relatively mild HL [42]. Regarding tinnitus severity, there was a significant association with increased anxiety, as reflected in higher scores on the HADS, highlighting the distinct yet overlapping effects of the classic MD symptomatic triad on patients’ overall well-being [28]. Interestingly, Stephens et al. demonstrated that, after controlling for the effects of other cardinal symptoms of MD, tinnitus primarily affected psychological domains, including anxiety, sleep disturbances, and depression, and also contributed to difficulties in listening situations and interactions with significant others [29]. Although tinnitus contributes substantially to disease-specific QoL, it shows little association with generic HRQoL measures, except that the mood domain is strongly linked to severe tinnitus [29].

Vestibular dysfunction can also contribute to cognitive impairments, particularly in visuospatial skills and executive function, further affecting daily functioning in patients with MD [43]. Their impact can be evaluated using complaint severity ratings, disease-specific measures, and impairment-related questionnaires [27].

These findings underscore the heterogeneity of MD presentation and highlight the potential influence of audiovestibular outcomes, psychological distress, and clinical progression on HRQoL. The primary objective of this observational, cross-sectional study is to address a critical gap by quantifying psychological distress and generic HRQoL between patients with definite MD and matched controls from the general population. Additionally, this study aims to examine whether increasing severity of HL, tinnitus, dizziness, and vertigo-related handicap is associated with differences in psychological distress (HADS) and generic HRQoL outcomes (SF-36 and EQ-5D-3L) within the MD cohort. To our knowledge, this is the first study to combine HADS, SF-36v2, EQ-5D-3L, THI, and DHI to compare patients with definite MD with a population-based Portuguese cohort and to disentangle the contributions of HL, tinnitus, and dizziness to psychological distress and HRQoL.

## 2. Materials and Methods

### 2.1. Study Design

This observational cross-sectional study compared psychological distress (HADS) and generic HRQoL (SF-36v2 and EQ-5D-3L) between patients with definite MD and matched controls from the general Portuguese population.

**Figure 1:**
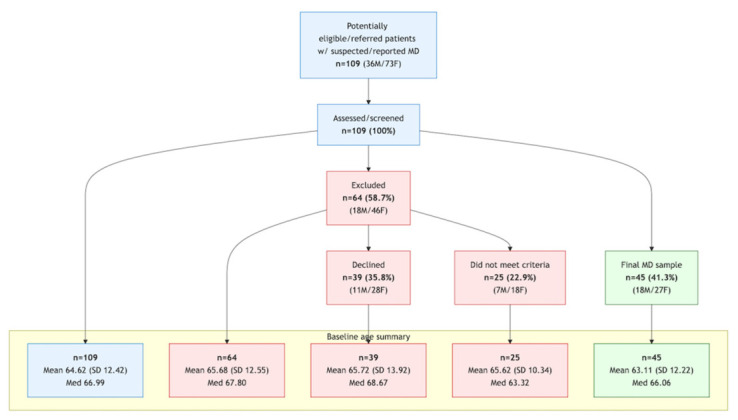
STROBE-compliant flowchart of participant selection and inclusion in the cross-sectional study.

Cases (*n* = 45) were clinic-based patients with confirmed definite MD, recruited from the otolaryngology department of a multispecialty medical clinic in Portugal. Between 2022 and 2024, 245 patients with MD were seen in the clinic. Using a prospective convenience sample, 45 adults who met the inclusion criteria and provided informed consent were enrolled between June 2024 and May 2025. All patients with MD were assessed during a clinically stable phase, outside of acute vertigo attacks. The recruitment flow is detailed in Figure 1, which presents a flowchart of participant selection and inclusion in accordance with the Strengthening the Reporting of Observational Studies in Epidemiology (STROBE) guidelines [44].

Controls (*n* = 135) were randomly selected from the EpiDoC cohort, a prospective, closed cohort population-based study of 10,661 non-institutionalized Portuguese adults (≥18 years) living in private households across mainland Portugal, Azores, and Madeira [45,46]. For this study, controls were drawn from the baseline evaluation of the EpiDoC 1 cohort (2011–2013), in which the same Portuguese versions of the SF-36v2, EQ-5D-3L, and HADS were administered using standardized interviewer-based protocols, comparable to those used in the MD group. Although 10-year intervals were used for practical feasibility, residual age-related confounding was assessed via continuous adjustment in sensitivity analyses, given the rarity of definite MD cases. This matching minimizes age- and sex-related differences, which are key determinants of HRQoL, while ensuring that controls are drawn from the community-dwelling population from which MD cases typically arise.

Within the MD group, we examined key clinical characteristics, including age at symptom onset, age at confirmed diagnosis, disease laterality (unilateral or bilateral), and disease-specific handicap (THI and DHI), in relation to psychological distress (HADS) and generic HRQoL (SF-36v2 and EQ-5D-3L). Pure-tone audiometry was performed on all participants to assess hearing thresholds, with pure-tone average (PTA) and severity staging calculated as described in the audiological assessment section.

This observational cross-sectional study was conducted in accordance with the STROBE guidelines [44].

### 2.2. Diagnostic Criteria

MD was diagnosed according to the 2015 consensus diagnostic criteria jointly formulated by the Classification Committee of the Bárány Society, Japan Society for Equilibrium Research, European Academy of Otology and Neurotology, Equilibrium Committee of the American Academy of Otolaryngology-Head and Neck Surgery (AAO-HNS), and Korean Balance Society [1].

### 2.3. Inclusion and Exclusion Criteria

Participants were formally recruited and assessed between June 2024 and May 2025 and included adults (≥18 years) with a definite diagnosis of MD according to the current diagnostic criteria. The exclusion criteria were as follows: inability to understand or provide informed consent; structural or pathological conditions of the external, middle, or inner ear; autoimmune or inflammatory inner ear disorders; central nervous system or vertebrobasilar cerebrovascular disorders; and other vestibular syndromes.

### 2.4. Audiological Assessment

Audiological assessment was performed to confirm the characteristic low- to mid-frequency SNHL required for a definite diagnosis of MD according to the Bárány Society criteria, while ruling out external or middle ear involvement [1]. The assessment followed international standards (ISO 8253-1:2010 [47] for procedures and ISO 389-1 [48] for calibration).

Otoscopy was performed to confirm the integrity of the external ear canal and tympanic membrane. Only participants with normal findings underwent further testing.

Tympanometry was performed using a GSI TympStar Pro immittance system with a variable air-pressure stimulus of a 226 Hz probe tone from +200 to −400 daPa. Tympanograms were classified according to the standard Jerger typology, and only type A was considered indicative of intact middle-ear function [49].

Pure-tone audiometry (air- and bone-conduction) was performed in a soundproof booth (IAC 401-A-SE) using a Grason-Stadler audiometer (Model GSI Pello) with RadioEar DD450 headphones and RadioEar B-81 bone-conduction transducer. Hearing thresholds were measured at frequencies ranging from 125 Hz to 8 kHz. PTA was calculated as the arithmetic mean of the thresholds at 500, 1000, 2000, and 3000 Hz [10]. Audiometric data were analyzed separately for the ears affected by MD (*n* = 52) and contralateral/unaffected ears (*n* = 38), enabling the characterization of hearing impairment in the MD cohort and comparison with unaffected ears within the same patients. HL severity was classified according to the 1995 AAO-HNS guidelines: stage 1 (PTA ≤ 25 dB HL), stage 2 (26–40 dB HL), stage 3 (41–70 dB HL), and stage 4 (>70 dB HL) [10]. For patients with bilateral MD, both affected ears were staged and analyzed separately, and no additional aggregation (e.g., worst-ear or averaged PTA) was performed at the patient level.

### 2.5. Questionnaires

The multidimensional impact of MD and related symptoms on patients’ daily functioning, psychological well-being, and overall HRQoL was thoroughly assessed using a standardized sociodemographic and clinical questionnaire, two disease-specific handicap questionnaires (DHI and THI), and three well-validated, culturally adapted patient-reported outcome measures: HADS for psychological distress, and SF-36v2 and EQ-5D-3L for generic HRQoL.

Sociodemographic characteristics (sex and age) and clinical history related to MD (age at symptom onset, age at confirmed diagnosis, and disease laterality) were collected using a study-specific standardized questionnaire developed for this project. A trained researcher administered the questionnaire in European Portuguese and recorded the responses in a secure electronic case report form to describe baseline characteristics.

Tinnitus severity was assessed using the European Portuguese version of the Tinnitus Handicap Inventory (THI-Pt) [50]. This validated 25-item questionnaire assesses tinnitus-related handicap across three subscales: functional (11 items), emotional (nine items), and catastrophic (five items) [50,51]. For statistical analysis, THI-Pt scores were dichotomized at a cutoff of >37, in accordance with the European Tinnitus Guidelines, grouping participants into “Mild Tinnitus” (slight and mild) and “Moderate-to-Severe Tinnitus” (moderate, severe, and catastrophic) [40].

Dizziness handicap was assessed using the European Portuguese version of the Dizziness Handicap Inventory (DHI-Pt) [52]. This validated 25-item questionnaire measures the impact of dizziness on daily functioning across three domains: physical (seven items), emotional (nine items), and functional (nine items) [52,53]. For statistical analysis, DHI-Pt total scores were dichotomized into “Mild Dizziness Handicap” (no handicap and mild) and “Moderate-to-Severe Dizziness Handicap” (moderate and severe).

Psychological distress was assessed using the European Portuguese version of the Hospital Anxiety and Depression Scale (HADS-Pt), a validated 14-item questionnaire that evaluates anxiety (HADS-A, seven items) and depression (HADS-D, seven items) symptoms in medical populations while minimizing the influence of physical illness symptoms [54,55].

HRQoL was assessed using the European Portuguese version of the Short Form 36-Item Health Survey, version 2 (SF-36v2-Pt), a validated 36-item questionnaire that evaluates eight health domains: physical functioning, role-physical, role-emotional, vitality, mental health, social functioning, bodily pain, and general health perceptions [56,57,58].

HRQoL was also assessed using the European Portuguese version of the EuroQol 5-Dimension 3-Level questionnaire (EQ-5D-3L-Pt), a validated instrument that evaluates five health dimensions: mobility, self-care, usual activities, pain/discomfort, and anxiety/depression [59]. Participants also rated their overall health status using the EQ-VAS, a visual analog scale ranging from zero (worst imaginable health) to 100 (best imaginable health). Utility scores were calculated using the Portuguese value set derived from time-trade-off valuations in the general population [60].

Of the 45 patients with MD, one had missing responses in selected patient-reported outcome measures (HADS-Pt, SF-36v2-Pt, EQ-5D-3L-Pt, THI-Pt, DHI-Pt), resulting in 44 valid questionnaires for those analyses. For each instrument, analyses were conducted on a complete-case basis (using all valid responses only).

### 2.6. Data Management

Data confidentiality was ensured through anonymization and secure storage in compliance with the General Data Protection Regulation (GDPR) and national legislation.

All data were entered into a secure electronic database with double-entry verification, regular backups, and restricted access, in accordance with the Good Clinical Practice guidelines.

### 2.7. Statistical Analysis

Statistical analyses were performed using descriptive statistics (mean and standard deviation [SD] for approximately normally distributed continuous variables, median and interquartile range [IQR] for non-normally distributed variables, and frequency and percentage for categorical variables). Group comparisons were conducted using independent *t*-tests or Mann–Whitney U tests for continuous variables, with the choice of parametric versus non-parametric tests based on the distribution of the data, and chi-square tests for categorical variables, as appropriate. Associations between HL stage and psychological distress and generic HRQoL outcomes were explored using one-way ANOVA or Kruskal–Wallis tests, followed by post hoc tests when indicated.

For the regression analyses, binary outcomes were defined as moderate-to-severe tinnitus handicap (THI-Pt > 37) and moderate-to-severe dizziness handicap (DHI-Pt ≥ 36) according to the established cutoffs. Univariable logistic regression models were fitted to examine crude associations between candidate predictors (age, sex, EQ-5D index, SF-36v2-Pt general health, HADS-A, and HADS-D) and each handicap outcome, and *p*-values for the regression coefficients were derived from this model. Variables with *p* < 0.10 in the univariable models and those deemed clinically relevant a priori were entered into multivariable logistic regression models, with adjustments for age and sex. Given that THI/DHI analyses were conducted within the MD cohort, standard binary logistic regression was deemed appropriate. Model results are reported as odds ratios (ORs) with corresponding 95% confidence intervals (95% CI) and *p*-values.

Given that matching was implemented at the frequency level (sex and 10-year age strata) rather than as fixed individual pairs, observations were treated as independent in the main analyses. As a sensitivity analysis, key between-group comparisons were re-evaluated in regression models adjusted for age and sex, which yielded similar results (available from the authors on request).

Given the exploratory nature of this study and the modest sample size, we performed multiple univariate comparisons across questionnaire domains without applying formal adjustments for multiple testing. Consequently, *p*-values should be interpreted with caution, with greater emphasis placed on the magnitude, consistency, and clinical plausibility of observed differences rather than on isolated borderline significances. All tests were two-tailed, and statistical significance was set at *p* < 0.05. Analyses were performed using SPSS version 28.0 (IBM Corp., Armonk, NY, USA).

## 3. Results

### 3.1. Participant Characteristics and Baseline Data

A total of 109 patients were identified as potentially eligible and referred for inclusion in the study. Following screening and assessment, 64 patients (58.7%) were excluded, primarily because of refusal to participate (*n* = 39), failure to meet the Bárány Society diagnostic criteria, or the presence of exclusion criteria (*n* = 25). The final study sample comprised 45 patients with definite MD, according to the Bárány Society criteria. Figure 1 presents a STROBE-compliant flowchart summarizing the participant selection process, including the reasons for exclusion and baseline age and sex distributions.

In the definite MD group (*n* = 45), the disease presentation was predominantly unilateral (*n* = 38; 84.4%), with the right ear affected in 21 cases (46.7%) and the left ear in 17 (37.8%); the remaining seven patients (15.6%) had bilateral involvement. The mean age at symptom onset was 48.56 (SD 14.23) years, and the definitive diagnosis was established at a mean age of 54.07 (SD 12.95) years, yielding a mean diagnostic delay of 5.51 (SD 4.89) years (range 0–19 years).

### 3.2. Comparison of Psychological Distress and HRQoL Between Patients with MD and EpiDoC Controls

Patients with definite MD showed a consistently less favorable generic HRQoL profile than age- and sex-matched controls from the Portuguese general population, as measured by the SF-36v2-Pt and EQ-5D-3L-Pt, and higher psychological distress on the HADS-Pt.

Owing to 1:3 frequency-matching strategy by sex and 10-year age strata, the definite MD group (*n* = 45) and the control group (*n* = 135) were perfectly balanced for sex (60.0% women in both groups) and highly comparable for age (mean 63.11, SD 12.22 years in cases vs. 62.64, SD 11.54 years in controls), effectively minimizing confounding by these major determinants of HRQoL. As expected from the frequency-matching strategy, there were no significant residual differences between patients with MD and controls in terms of age distribution or sex (*p* = 0.659 for age and *p* = 1.000 for sex, Mann–Whitney U and chi-square tests, respectively). In age- and sex-adjusted sensitivity analyses using regression models, the associations between MD status and HADS-Pt, SF-36v2-Pt, and EQ-5D-3L-Pt outcomes were very similar to the unadjusted comparisons, with only minimal changes in effect sizes and *p*-values and no change in the direction or statistical significance of the main findings (results available from the authors on request).

#### 3.2.1. HADS-Pt

In the HADS-Pt, patients with MD (*n* = 44 valid responses) had significantly higher depression scores than controls (median 6.00 [IQR 2.00–8.75] vs. 3.00 [IQR 1.00–6.00]; *p* = 0.004, Mann–Whitney U test) and higher anxiety scores (median 8.00 [IQR 6.00–11.00] vs. 5.00 [IQR 2.00–9.00]; *p* < 0.001, Mann–Whitney U test), indicating a substantially higher burden of psychological distress in patients with MD. Although the proportion of abnormal depression scores did not differ significantly between groups (15.9% vs. 10.4%; *p* = 0.417, chi-square test), abnormal anxiety scores were almost twice as frequent in the MD cohort (29.5% vs. 14.1%; *p* = 0.025, chi-square test).

**Figure 2:**
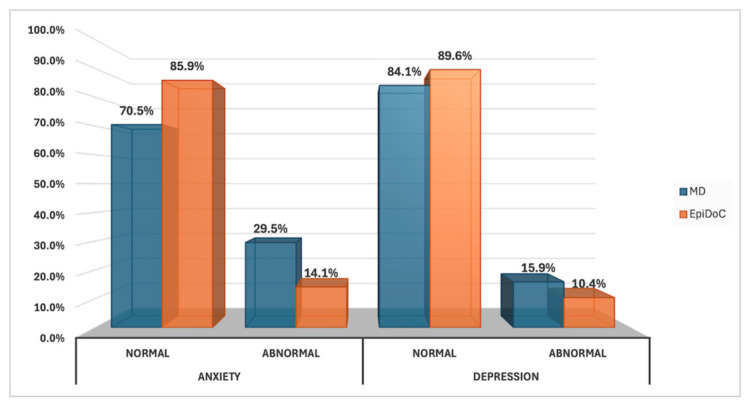
Proportion of normal and abnormal scores on the HADS-Pt anxiety and depression subscales in patients with MD (blue; *n* = 44) compared with the EpiDoC population-based sample (orange; *n* = 135). Percentages are labeled directly on the bars. Abbreviations: HADS-Pt, Hospital Anxiety and Depression Scale—Portuguese version; MD, Ménière’s disease.

Figure 2 presents the descriptive frequencies (percentages) of normal and abnormal scores on the HADS-Pt anxiety and depression subscales among patients with MD compared with the EpiDoC population-based sample.

#### 3.2.2. SF-36v2-Pt

Table 1 presents the SF-36v2-PT domain scores for both groups. In patients with MD (*n* = 44 valid responses per domain), the mean values ranged from 51.02 (general health) to 69.51 (role-emotional). In the EpiDoC sample (*n* = 135), the mean values ranged from 55.00 (bodily pain) to 83.61 (social functioning).

The SF-36v2-Pt results revealed a consistent pattern of impairment across most domains in patients with MD. Compared with the EpiDoC controls, patients with MD had significantly worse role-physical scores (median 75.00 vs. 100.00; *p* < 0.001), role-emotional scores (75.00 vs. 100.00; *p* < 0.001), vitality (50.00 vs. 65.00; *p* = 0.004), mental health (57.50 vs. 64.00; *p* = 0.036), and social functioning (75.00 vs. 100.00; *p* < 0.001). Notably, patients with MD reported significantly less bodily pain than controls (67.50 vs. 50.00; *p* = 0.022), whereas physical functioning (67.50 vs. 85.00; *p* = 0.173) and general health (50.00 vs. 55.00; *p* = 0.110) did not differ significantly between the groups. All comparisons were performed using the Mann–Whitney U test.

#### 3.2.3. EQ-5D-3L-Pt

**Figure 3:**
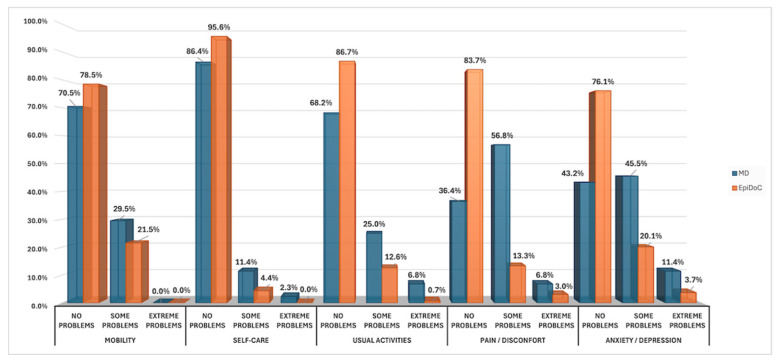
Distribution of responses across the five EQ-5D-3L-Pt dimensions in patients with MD (blue; *n* = 44) compared with the EpiDoC population-based sample (orange; *n* = 135). The bars represent the percentage of respondents who reported no, some, or extreme problems in each dimension. Abbreviations: EQ-5D-3L-Pt, EuroQol 5-Dimension 3-Level questionnaire; MD, Ménière’s disease.

Figure 3 shows the distribution of responses across the three EQ-5D-3L-Pt levels (no problems, some problems, and extreme problems) for patients with MD (*n* = 44 valid responses) compared with a representative EpiDoC population sample.

EQ-5D-3L-Pt profiles highlighted specific problem areas. Patients with MD reported a much higher prevalence of pain/discomfort (only 36.4% “no problems” vs. 83.7% in controls; *p* < 0.001, chi-square test) and anxiety/depression (43.2% “no problems” vs. 76.1%; *p* < 0.001, chi-square test) than controls. Although differences in mobility, self-care, and usual activities did not reach statistical significance, the EQ-5D index score was markedly lower in the MD cohort than in the controls (median 0.66 [IQR 0.45–0.94] vs. 1.00 [IQR 0.69–1.00]; *p* < 0.001, Mann–Whitney U test), indicating a substantial reduction in overall health utility associated with MD.

### 3.3. Associations Within Patients with MD

To further characterize HRQoL in the MD group, we examined the associations of THI-Pt, DHI-Pt, and HL stage with psychological distress and HRQoL outcomes.

#### 3.3.1. HL Severity and HRQoL Outcomes

**Figure 4:**
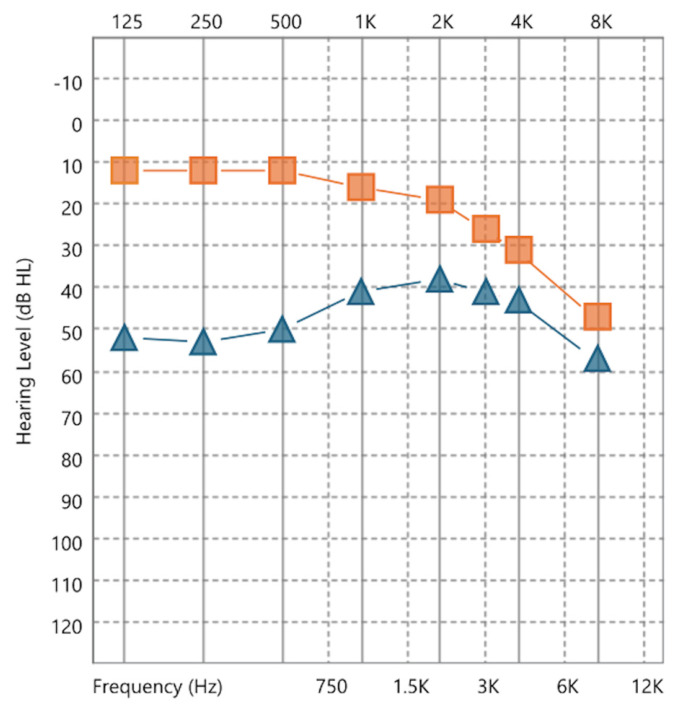
Mean pure-tone hearing thresholds (dB HL) for ears affected by MD (blue) compared with contralateral unaffected ears (orange) in 45 patients (52 affected ears and 38 unaffected ears; 38 unilateral [22 right, 17 left] and seven bilateral). Abbreviations: MD, Ménière’s disease; dB HL, decibels hearing level.

HL assessment revealed significant impairment in the MD-affected ears. Pure-tone audiometry was performed in 45 patients, yielding 52 affected ears (38 unilateral [22 right, 17 left] and seven bilateral) and 38 unaffected (contralateral) ears. Consistent with the audiological assessment, HL severity analyses were conducted at the ear level, including both MD-affected ears in bilateral cases. The mean PTA was 42.26 dB HL (SD 23.78) in the affected ears and 18.02 dB HL (SD 12.41) in the contralateral ears. PTA classification according to the 1995 AAO-HNS criteria in Ménière’s ears showed the following distribution: 16 (30.8%) stage 1, 14 (26.9%) stage 2, 14 (26.9%) stage 3, and 8 (15.4%) stage 4. Figure 4 shows an audiogram illustrating the mean hearing thresholds across frequencies (125 Hz to 8 kHz) for Ménière’s ears (blue) versus contralateral ears (orange). Table 2 presents the mean (SD) thresholds and number of valid measurements at key frequencies (500, 1000, 2000, and 3000 Hz), along with the corresponding PTA values.

Within the MD cohort, HADS-D scores increased modestly from stages 1 to 4 (mean 4.82, SD 3.71; 6.00, SD 5.31; 5.23, SD 3.86; and 7.88, SD 2.70, respectively). However, these differences were not statistically significant (*p* = 0.412, ANOVA). The HADS-A scores were also comparable across stages (mean 8.82, SD 3.66; 8.58, SD 4.81; 7.69, SD 3.30; and 7.88, SD 3.76; *p* = 0.884, ANOVA), and the categorical distributions of normal vs. abnormal depression and anxiety did not permit robust chi-square testing.

SF-36v2-Pt domain scores showed no statistically significant differences across stages (all *p* > 0.05, Kruskal–Wallis). Physical functioning scores were higher in stages 1 and 2 than in stages 3 and 4 (median 85 [IQR 65–95] and 90 [IQR 55–98.75] vs. 55 [IQR 32.50–82.50] and 55 [IQR 51.25–83.75]; *p* = 0.092, Kruskal–Wallis), while other domains (role-physical, role-emotional, vitality, mental health, social functioning, bodily pain, and general health) varied only slightly between stages.

The problem levels across the five dimensions of the EQ-5D-3L-Pt were broadly similar across stages, and the chi-square tests were limited by sparse cells. EQ-5D index values did not differ significantly by hearing stage (mean 0.667, 0.600, 0.657, and 0.520 for stages 1–4; *p* = 0.694, ANOVA).

#### 3.3.2. THI-Pt and HRQoL Outcomes

The THI-Pt was completed by 44 patients (one participant had missing data), with a mean total score of 44.91 (SD 26.81). According to the standard THI grading system, the severity distribution was as follows: slight or no handicap (*n* = 7, 15.9%), mild (*n* = 11, 25.0%), moderate (*n* = 10, 22.7%), severe (*n* = 13, 29.5%), and catastrophic (*n* = 3, 6.8%). Eighteen patients (40.9%) were classified as having mild tinnitus, and 26 (59.1%) as having moderate-to-severe tinnitus.

Tinnitus severity was significantly associated with both depression and anxiety scores on the HADS-Pt. Patients with moderate-to-severe tinnitus had significantly higher mean depression scores (7.00, SD 4.30) than those with mild tinnitus (4.11, SD 3.22; *p* = 0.020, Student’s *t*-test). Similarly, mean anxiety scores were significantly higher in the moderate-to-severe group (9.69, SD 3.95) than in the mild group (6.17, SD 2.50; *p* = 0.002, Student’s *t*-test). Categorical analyses revealed a significant association with the proportion of abnormal scores. Regarding depression, all patients with mild tinnitus had normal scores (100%), whereas 26.9% of those with moderate-to-severe tinnitus had abnormal scores (*p* = 0.031, chi-squared test). Similarly, for anxiety, only 5.6% of the mild group had abnormal scores compared with 46.2% of the moderate-to-severe group (*p* = 0.006, chi-square test).

Patients with mild tinnitus handicap had significantly higher SF-36v2-Pt scores than those with moderate-to-severe tinnitus across most domains. Specifically, significant differences were observed in physical functioning (median 85 [IQR 68.75–100] vs. 55 [IQR 42.50–90.00]; *p* = 0.031), role-physical (87.50 [IQR 60.94–100.00] vs. 53.13 [IQR 35.94–89.06]; *p* = 0.045), role-emotional (95.83 [IQR 64.58–100] vs. 58.33 [IQR 31.25–93.75]; *p* = 0.015), mental health (64.17 ± 11.28 vs. 52.69 ± 15.70; *p* = 0.011), social functioning (87.50 [IQR 71.88–100] vs. 62.50 [IQR 37.50–75.00]; *p* = 0.013), and bodily pain (83.75 [IQR 52.50–100] vs. 60.00 [IQR 45.00–69.38]; *p* = 0.046, all Mann–Whitney U tests, except for mental health, Student’s *t*-test). No significant differences were found in vitality (median 54.17, SD 19.76 vs. 49.76, SD 21.83; *p* = 0.498, Student’s *t*-test) or general health (55.28, SD 19.51 vs. 48.08, SD 19.29; *p* = 0.232, Student’s *t*-test).

Across the EQ-5D-3L-Pt dimensions, the distribution of problems was broadly similar between patients with mild and moderate-to-severe tinnitus. In both groups, most patients reported no problems in mobility, self-care, and usual activities, whereas some degree of limitation was more frequent in pain/discomfort and anxiety/depression in the higher severity group. However, chi-square tests were unreliable owing to sparse cells. Consistently, there was no statistically significant association between tinnitus severity and the EQ-5D index score (median 0.69 [IQR 0.53–1.00] vs. 0.56 [IQR 0.32–0.77]; *p* = 0.132, Mann–Whitney U test).

#### 3.3.3. DHI-Pt and HRQoL Outcomes

Dizziness-related handicap was assessed using the DHI-Pt in the same 44 patients with available data. The mean total score was 36.45 (SD 20.50). The domain-specific mean scores were as follows: functional, 14.14 (SD 8.36); emotional, 11.95 (SD 7.82); and physical, 10.36 (SD 5.68). According to the DHI severity classification, six patients (13.6%) had no handicap, 14 (31.8%) had a mild handicap, 17 (38.6%) had a moderate handicap, and seven (15.9%) had a severe one. This resulted in 20 patients (45.5%) classified as having mild dizziness handicap and 24 patients (54.5%) as having moderate-to-severe dizziness handicap.

Dizziness- and vertigo-related handicap were significantly associated with depression and anxiety scores on the HADS-Pt. Patients with moderate-to-severe dizziness handicap had significantly higher median depression scores (8.00 [IQR 6.00–11.00]) than those with mild dizziness handicap (2.50 [IQR 0.25–4.75]; *p* < 0.001, Mann–Whitney U test). Similarly, the mean anxiety scores were significantly higher in the moderate-to-severe dizziness handicap group (9.58, SD 3.96) than in the mild dizziness handicap group (6.65, SD 3.03; *p* = 0.010, Student’s *t*-test). Categorical analysis further revealed significant associations with the proportion of abnormal scores. For depression, all patients with mild dizziness handicap (*n* = 20) had normal scores (100%), whereas 29.2% of those with moderate-to-severe dizziness handicap (*n* = 24) had abnormal scores (*p* = 0.011; chi-squared test). For anxiety, 90.0% of the mild dizziness handicap group had normal scores (10.0% abnormal), compared with 54.2% normal scores in the moderate-to-severe dizziness handicap group (45.8% abnormal; *p* = 0.018, chi-squared test).

Patients with mild dizziness-related handicap had markedly higher SF-36v2-Pt scores than those with moderate-to-severe dizziness-related handicap across nearly all domains. Statistically significant differences were observed in physical functioning (median 87.50 [IQR 57.50–100] vs. 60.00 [IQR 41.25–85.00]; *p* = 0.018), role physical (96.88 [IQR 75.00–100] vs. 50.00 [IQR 25.00–79.69]; *p* < 0.001), role emotional (100.00 [IQR 77.08–100] vs. 54.17 [IQR 25.00–79.17]; *p* < 0.001), vitality (59.38, SD 22.63 vs. 45.05, SD 17.19; *p* = 0.022), mental health (63.75, SD 12.66 vs. 52.08, SD 15.03; *p* = 0.009), social functioning (100 [IQR 75.00–100] vs. 56.25 [IQR 37.50–75.00]; *p* < 0.001), and general health (61.75, SD 19.08 vs. 42.08, SD 15.03; *p* < 0.001). Mann–Whitney U tests were used for physical, social, and role domains, and Student’s *t*-test for vitality, mental health, and general health. Bodily pain showed no significant difference in the DHI-Pt comparison (median 72.50 [IQR 55.00–100.00] vs. 60.00 [IQR 45.00–73.13]; *p* = 0.109, Mann–Whitney U test).

Similar to the THI-PT, EQ-5D-3L-Pt dimension profiles did not differ markedly between patients with mild and moderate-to-severe dizziness handicap, with most participants in both groups reporting no problems in mobility, self-care, or usual activities. Reports of pain/discomfort and anxiety/depression were more common in the moderate-to-severe dizziness group. However, formal categorical comparisons were limited by small cell counts. There was no statistically significant association between dizziness- and vertigo-related handicap and the EQ-5D index score (mean 0.68, SD 0.29 vs. 0.57, SD 0.29; *p* = 0.251, Student’s *t*-test).

### 3.4. Regression Models

#### 3.4.1. THI-Pt

In the multivariable logistic regression model for tinnitus handicap (*n* = 44; 26 with moderate-to-severe THI-Pt), abnormal anxiety emerged as the only independent predictor after adjustment for age and sex. Patients with abnormal anxiety had markedly higher odds of moderate-to-severe tinnitus handicap than those with normal anxiety (OR 17.51, 95% CI 1.86–165.35; *p* = 0.012). Sex and age were not significantly associated with THI-Pt severity (OR 1.59, 95% CI 0.37–6.89; *p* = 0.537, and OR 0.99, 95% CI 0.94–1.05; *p* = 0.785, respectively). Although higher depression scores were significantly associated with moderate-to-severe tinnitus in univariable analyses, this association was attenuated and no longer statistically significant when anxiety was included in the multivariable model, suggesting substantial overlap between depressive and anxiety symptoms in relation to tinnitus-related handicap. Considering the modest sample size and number of events, these findings should be interpreted as exploratory and hypothesis-generating.

#### 3.4.2. DHI-Pt

In the multivariable model for dizziness handicap (*n* = 44; 24 with moderate-to-severe DHI-Pt), higher depressive symptoms and abnormal anxiety were associated with increased odds of moderate-to-severe dizziness handicap after adjustment for age and sex. Each one-point increase in the HADS-D score was associated with a 64% increase in the odds of moderate-to-severe dizziness handicap (OR 1.64 per point, 95% CI 1.21–2.23; *p* = 0.001). Abnormal anxiety showed a strong but borderline association with moderate-to-severe dizziness handicap (OR 11.74, 95% CI 0.95–145.28; *p* = 0.055). Sex and age were not significantly associated with DHI-Pt severity (OR 1.16, 95% CI 0.18–7.52; *p* = 0.879, and OR 1.03, 95% CI 0.96–1.12; *p* = 0.415, respectively). In univariable analyses, worse general health (SF-36v2-Pt) was significantly associated with higher DHI-Pt scores. Still, this effect was attenuated and no longer statistically significant in the multivariable model, consistent with partial mediation through psychological distress. Given the limited number of events and the dichotomization of THI/DHI, these regression findings should also be viewed as exploratory.

## 4. Discussion

### 4.1. Comparison of HRQoL Between Patients with MD and EpiDoC Controls

In this matched case–control study, patients with definite MD had a consistently less favorable generic HRQoL profile and a substantially higher burden of psychological distress than age- and sex-matched adults from the EpiDoC cohort, confirming that MD exerts a substantial psychosocial burden beyond what would be expected for age alone [28,38]. Higher HADS-Pt anxiety and depression scores, together with the increased proportion of abnormal anxiety in the MD group, are in line with prior reports that psychological distress is a common and clinically relevant component of MD, driven by the unpredictability of vertigo attacks and the chronicity of tinnitus and HL [29,31].

The pattern of the SF-36v2-Pt results partly reflects the complex interplay between the classic MD triad and generic functioning, as described in a previous study [28]. Söderman et al. reported that vertigo primarily impairs the physical aspects of HRQoL, whereas HL and tinnitus affect psychosocial domains, including mood and social interactions [28]. Simultaneously, Stephens et al. emphasized the psychological and relational impacts of tinnitus after adjusting for other MD symptoms [29]. In our study, patients with MD showed consistently worse scores than the EpiDoC controls, indicating a broad impact of MD on both physical role performance and psychosocial domains. Notably, patients with MD reported less bodily pain than controls, which may reflect differences in non-MD comorbidities and musculoskeletal conditions in the community-dwelling EpiDoC sample, rather than a genuine protective effect of MD on pain. The marked reduction in EQ-5D index values and the higher frequency of pain/discomfort and anxiety/depression in patients with MD reinforce that, at the population level, MD is associated with a meaningful loss of health utility, consistent with earlier studies showing impaired generic HRQoL in vestibular and audiological disorders [30,38].

From an audiology/otology perspective, these results indicate that routine MD follow-up should not be limited to audiometric assessments. Still, they should also include systematic screening for anxiety and depression using brief validated tools, such as the HADS. Incorporating a generic HRQoL measure (e.g., EQ-5D or SF-36) can further help quantify the overall burden and facilitate patient-centered monitoring over time.

### 4.2. Associations Within Patients with MD

Within the MD cohort, HL severity showed only weak, non-significant associations with generic HRQoL and psychological distress in ear-level analyses. Given that the HL stage was defined at the ear level, including both affected ears in bilateral cases, whereas HRQoL and psychological distress were assessed at the patient level, these results should be interpreted cautiously because of a residual unit-of-analysis limitation. This pattern is nonetheless compatible with previous studies, in which audiometric thresholds alone do not fully capture the lived impact of MD, which depends on fluctuation, laterality, and accompanying vestibular and tinnitus symptoms [28,29,43]. In clinical practice, this underscores that patients with relatively mild audiograms may still report significant psychosocial impairment, whereas some with more advanced loss adapt better than expected [5,42]. These observations support the notion that audiometric measures alone may underestimate the lived impact of MD-related tinnitus and HL on psychological distress and HRQoL, and that disease-specific handicap instruments capture additional clinically important variance [5,28,29,42,43].

In contrast, disease-specific handicap measures were closely aligned with the profiles described by Söderman and Stephens [28,29]. Higher tinnitus handicap (THI-Pt) was associated with more severe anxiety and depression symptoms on the HADS-Pt and with worse SF-36v2-Pt scores in several physical, role, social, and emotional domains, mirroring Stephens et al.’s observation that tinnitus is tightly linked to anxiety, sleep disturbance, and depressive symptoms, even when generic HRQoL instruments sometimes appear less sensitive to tinnitus alone [29]. Dizziness- and vertigo-related handicap (DHI-Pt) showed an even broader and stronger relationship with psychological distress and HRQoL, with moderate-to-severe dizziness associated with higher psychological distress and lower SF-36v2-Pt scores across nearly all domains, consistent with Söderman et al.’s finding that vertigo is a key driver of physical HRQoL limitations and functional restriction [28,61]. The absence of statistically significant gradients in EQ-5D index values by THI-Pt or DHI-Pt suggests that while EQ-5D is useful for capturing global health utility differences between MD and the general population, it may be less sensitive to within-cohort variations in specific audiovestibular disabilities [27,38].

The regression models suggested that psychological distress, particularly anxiety, is a prominent correlate of disease-specific handicap in MD and may represent a modifiable clinical target, although causal inferences cannot be drawn from this cross-sectional design. Patients with abnormal anxiety had markedly higher odds of moderate-to-severe tinnitus handicap (approximately 17-fold) and showed a strong but borderline trend toward higher odds of moderate-to-severe dizziness handicap, independent of age and sex, with wide confidence intervals reflecting the small number of events, underscoring the bidirectional relationship between vestibular symptoms and mental health, as previously reported by Weidt et al. and Porter & Boothroyd [30,62].

Notably, depression independently predicted dizziness handicap (approximately a 60% increased risk per HADS-D point), whereas anxiety dominated the effect for tinnitus handicap, consistent with Söderman et al.’s finding that tinnitus severity correlates specifically with HADS-A [28]. These findings suggest that targeted anxiety management, alongside depression screening, may be critical to reducing symptom burden in MD and should complement vestibular rehabilitation and audiological interventions.

Beyond screening, the combined use of the THI/DHI, HADS, and a generic HRQoL measure in otology/audiology clinics may also improve clinical stratification for referral (e.g., vestibular rehabilitation and mental health support) and follow-up prioritization in routine care.

## 5. Conclusions

Definite MD is associated with clinically important decrements in generic HRQoL and psychological distress compared with age- and sex-matched adults from the Portuguese general population, with consistently worse SF-36v2-Pt scores in role and psychosocial domains, markedly lower EQ-5D index values, and higher levels of pain/discomfort and anxiety/depression.

Within the MD cohort, audiometric HL severity was only weakly related to generic HRQoL and psychological distress. In contrast, tinnitus- and dizziness-related handicap emerged as the main correlates of poorer functioning, supporting the use of disease-specific instruments (THI-Pt and DHI-Pt) alongside conventional audiometry in clinical assessment. Furthermore, logistic regression models suggested that psychological distress, particularly anxiety and, to a lesser extent, depression, is a prominent correlate and plausible modifiable target in relation to disease-specific handicap, with abnormal anxiety associated with markedly increased odds of moderate-to-severe tinnitus handicap and a strong but borderline association with dizziness handicap, while higher depressive symptoms independently predicted the latter, although causal inferences cannot be drawn from this cross-sectional design.

These findings support a symptom-focused multidisciplinary management strategy that, in addition to standard audiological care, prioritizes tinnitus- and dizziness-related handicap and psychological distress, particularly anxiety, as potential targets to improve HRQoL in patients with MD. Accordingly, future interventional and longitudinal studies on MD should include psychological distress, THI/DHI, and generic HRQoL as core endpoints to better quantify patient-centered benefits beyond audiometric changes and to clarify the directionality of these associations and inform multidisciplinary care pathways.

### Limitations

First, MD cases were based on clinical visits. They included patients with definite disease recruited from the ENT department of a multispecialty medical clinic in Portugal. In contrast, controls were population-based participants from the EpiDoC cohort, a classic but unavoidable design feature in rare disease research that may introduce spectrum bias, as clinic patients are likely to represent more severe or refractory MD than undiagnosed or less symptomatic cases in the community. Second, the recruitment periods differed: patients with MD were diagnosed and followed in this ENT department between 2022 and 2024 and were enrolled in this study between June 2024 and May 2025, whereas controls were drawn from the baseline evaluation of the EpiDoC 1 cohort (2011–2013). Therefore, subtle temporal changes in population HRQoL norms (e.g., post-pandemic effects) cannot be entirely ruled out. Third, the modest sample size and the distribution of patients across HL and handicap strata led to small cell counts in several subgroups, particularly for some EQ-5D-3L-Pt dimensions. In addition, the HL stage was defined at the ear level, including both affected ears in bilateral cases, whereas HRQoL, psychological distress, tinnitus, and dizziness handicap were assessed at the patient level. This mismatch in the unit of analysis introduces residual dependence between observations from the same patient and limits the precision with which we can characterize the relationship between HL stage and patient-reported outcomes. Therefore, conclusions regarding the weak association between HL stage and HRQoL/psychological distress should be regarded as cautious and exploratory. However, these numbers are comparable to other single-center studies of rare conditions in countries with smaller populations, such as Portugal, and still allowed us to detect clinically meaningful between-group differences, although the power to detect subtle within-cohort gradients was limited. In addition, because many univariate comparisons were performed across several HRQoL and handicap domains, the risk of inflated type I error due to multiple testing cannot be excluded; as a result, some statistically significant findings, particularly those with *p*-values close to 0.05, should be interpreted cautiously and regarded as hypothesis-generating. Finally, the cross-sectional design precluded causal inference and did not capture the fluctuating nature of MD symptoms over time. Moreover, we did not systematically capture the time since the last vertigo attack or formal stages of disease activity (“burnt-out” versus active MD), which limits our ability to explore how acute versus quiescent phases might differentially affect HRQoL. Although all HRQoL and handicap instruments were validated in Portuguese, their self-reported nature introduces the possibility of reporting bias and residual confounding from unmeasured clinical and socioeconomic factors.

## Figures and Tables

**Table 1:** SF-36v2-Pt domain scores in patients with MD compared with those in the EpiDoC population-based sample. Data are presented as median and IQR. All between-group comparisons were performed using the Mann–Whitney U test. Abbreviations: MD, Ménière’s disease; IQR, interquartile range; SF-36v2-Pt, Short Form-36 version 2 Portuguese.

Domain	MD Sample (*n* = 44)		EpiDoC Sample (*n* = 135)		*p* Value
	Median	IQR (Q1–Q3)	Median	IQR (Q1–Q3)	
Physical functioning	67.50	50.00–90.00	85.00	50.00–100.00	0.173
Role-Physical	75.00	43.75–100.00	100.00	75.00–100.00	<0.001
Bodily Pain	67.50	45.00–97.50	50.00	50.00–67.50	0.022
General health	50.00	35.00–65.00	55.00	45.00–70.00	0.110
Vitality	50.00	31.25–68.75	65.00	45.00–80.00	0.004
Social functioning	75.00	50.00–100.00	100.00	75.00–100.00	<0.001
Role-Emotional	75.00	50.00–100.00	100.00	66.67–100.00	<0.001
Mental Health	57.50	50.00–70.00	64.00	48.00–84.00	0.036

**Table 2:** Pure-tone hearing thresholds and PTA in ears affected by MD versus contralateral unaffected ears. Data are presented as mean (SD). HL severity was staged according to the 1995 AAO-HNS criteria. Note: n varied slightly at 3000 Hz owing to missing measurements in some ears. Abbreviations: MD, Ménière’s disease; PTA, pure-tone average; dB HL, decibels hearing level; AAO-HNS, American Academy of Otolaryngology-Head and Neck Surgery.

Ears	Frequencies												PTA		
	500 Hz			1000 Hz			2000 Hz			3000 Hz					
	Mean	SD	[*n*]	Mean	SD	[*n*]	Mean	SD	[*n*]	Mean	SD	[*n*]	Mean	SD	[*n*]
MD	49.71	22.63	52	41.44	25.43	52	37.5	26.09	52	41.63	25.56	49	42.26	23.78	52
Contralateral	11.84	9.69	38	16.05	11.69	38	19.08	15.46	38	25.51	18.32	37	18.02	12.41	38

## Data Availability

The data presented in this study are available upon request from the corresponding author due to privacy and data protection restrictions in accordance with the General Data Protection Regulation (GDPR).

## Abbreviations

The following abbreviations are used in this manuscript:

MD	Ménière’s Disease
SNHL	Sensorineural Hearing Loss
HL	Hearing Loss
QoL	Quality of Life
HRQoL	Health-Related Quality of Life
AAO-HNS	American Academy of Otolaryngology-Head and Neck Surgery
HADS	Hospital Anxiety and Depression Scale
HADS-Pt	European Portuguese version of the Hospital Anxiety and Depression Scale
SF-36v2	Short Form 36-Item Health Survey, version 2
SF-36v2-Pt	European Portuguese version of the Short Form 36-Item Health Survey, version 2
EQ-5D-3L	EuroQol 5-Dimension 3-Level questionnaire
EQ-5D-3L-Pt	European Portuguese version of the EuroQol 5-Dimension 3-Level questionnaire
THI	Tinnitus Handicap Inventory
THI-Pt	European Portuguese version of the Tinnitus Handicap Inventory
DHI	Dizziness Handicap Inventory
DHI-Pt	European Portuguese version of the Dizziness Handicap Inventory
PTA	Pure-Tone Average
SD	Standard Deviation
IQR	Interquartile Range
OR	Odds Ratio
STROBE	Strengthening the Reporting of Observational Studies in Epidemiology
